# Non-Coding RNAs Associated With Radioresistance in Triple-Negative Breast Cancer

**DOI:** 10.3389/fonc.2021.752270

**Published:** 2021-11-04

**Authors:** Alberto Aranza-Martínez, Julio Sánchez-Pérez, Luis Brito-Elias, César López-Camarillo, David Cantú de León, Carlos Pérez-Plasencia, Eduardo López-Urrutia

**Affiliations:** ^1^ Laboratorio de Genómica Funcional, Facultad de Estudios Superiores Iztacala Universidad Nacional Autónoma de México (UNAM), Tlalnepantla, Mexico; ^2^ Posgrado en Ciencias Genómicas, Universidad Autónoma de la Ciudad de México, Mexico City, Mexico; ^3^ Dirección de Investigación, Instituto Nacional de Cancerología (INCan), Mexico City, Mexico; ^4^ Laboratorio de Genómica, Instituto Nacional de Cancerología (INCan), Mexico City, Mexico

**Keywords:** breast cancer, triple negative breast-cancer, radioresistance, non-coding RNAs, long non-coding RNAs, microRNAs

## Abstract

The resistance that Triple-Negative Breast Cancer (TNBC), the most aggressive breast cancer subtype, develops against radiotherapy is a complex phenomenon involving several regulators of cell metabolism and gene expression; understanding it is the only way to overcome it. We focused this review on the contribution of the two leading classes of regulatory non-coding RNAs, microRNAs (miRNAs) and long non-coding RNAs (lncRNAs), against ionizing radiation-based therapies. We found that these regulatory RNAs are mainly associated with DNA damage response, cell death, and cell cycle regulation, although they regulate other processes like cell signaling and metabolism. Several regulatory RNAs regulate multiple pathways simultaneously, such as miR-139-5p, the miR-15 family, and the lncRNA HOTAIR. On the other hand, proteins such as CHK1 and WEE1 are targeted by several regulatory RNAs simultaneously. Interestingly, the study of miRNA/lncRNA/mRNA regulation axes increases, opening new avenues for understanding radioresistance. Many of the miRNAs and lncRNAs that we reviewed here can be used as molecular markers or targeted by upcoming therapeutic options, undoubtedly contributing to a better prognosis for TNBC patients.

## Introduction

Breast cancer (BC) is the malignant tumor with the highest number of cases diagnosed worldwide and the most common cause of death in women (1). Although it is a heterogeneous disease, breast tumors can be classified based on the expression level of hormonal receptors for estrogen (ER), progesterone (PR), and human epidermal growth factor receptor 2 (HER2) in subtypes depending on the presence (+) or absence (–) of hormonal receptors, namely Luminal A (ER+, PR+/-, HER2-), Luminal B (ER+, PR+/-, HER2+), and HER2-enriched (ER -, PR -, HER2+). A fourth subtype that lacks the expression of all the mentioned hormonal receptors is named Triple Negative (ER -, PR -, HER2-) or Basal-like (2).

Triple-Negative Breast Cancer (TNBC) is further classified into four subtypes; Basal-like 1 (BL1), Basal-like 2 (BL2), Mesenchymal (M), and Luminal Androgen Receptor positive (LAR), where each subtype considers cancerous stage, gene pattern expression, propagation, metastasis, histologic differences and response to common chemotherapeutic neoadjuvants (3). Among the breast cancer subtypes, TNBC is the most aggressive, has a poor prognosis and a high risk of recurrence and metastasis (4–6), and complicates targeted therapies in patients due to the absence of hormonal receptors (ER, PR, HER2) (6).

TNBC patients commonly receive systemic treatments such as chemotherapy or local therapies, including conventional surgery and radiotherapy either in isolation or in combination with other types of treatments for increased effectiveness and prognosis after surgery (6–9).

Radiotherapy has proved efficient for breast cancer patients after mastectomy, at least in levels I and II, reducing recurrence and mortality (10). This type of therapy employs ionizing radiation (IR), e.g., X-rays, gamma rays, α, and β particles, ion carbon or electron, neutron, and proton beams (11, 12) to improve the diagnosis.

IR affects cells directly and indirectly. The direct effect is promoting DNA damage like single-strand breaks (SSBs), double-strand breaks (DSBs), also called clustered DNA lesions, genomic instability, and inducing apoptosis. On the other hand, the indirect effect is caused by reactive oxygen species (ROS) generated from the interconnection between IR and water, promoting complex DNA lesions that alter cell homeostasis, modifying proteins and lipids, eventually lead to cell death (13–15). Nevertheless, the implementation of radiotherapy is still controversial (16), and its efficacy may be limited by the presence of tumor cells resistant to ionizing radiation (17) due to alterations in the pathways and genes involved in the DNA damage response system (DDR).

The alteration of these elements that generally play an important role in preserving cell viability through the repairing genetic material modifies the response of tumor cells to radiotherapy (18).

Recently, it has been observed that not only the irradiated cells themselves react by modifying their metabolism, but that they communicate with neighboring, unirradiated cells through gap junctions and secreted small molecules in a mechanism known as ‘radiation-induced bystander effect’ (RIBE). Through RIBE, bystander cells can rescue irradiated cells, increasing their survival (19). It has been shown that angiogenesis, invasion, metastasis, and proliferative signaling maintenance can also be induced through RIBE, affecting the outcome of IR therapies and enhancing radioresistance (20).

Several groups have reported mechanisms that lead cells to resistance to TNBC therapies, such as hypoxia, cell cycle regulation (21), signaling pathways linked to radiosensitivity like mTOR (22) and EGFR/PI3K/Akt (23), among others. Here we want to highlight the role of two classes of non-coding RNAs (ncRNAs), microRNAs (miRNAs) and long non-coding RNAs (lncRNAs), in the development of radioresistance.

MicroRNAs are small, 21-25 nucleotide-long, single-stranded RNA molecules (24) that negatively regulate mRNA through binding their 3’ UTR and blocking translation (25, 26). They are involved in virtually every cellular process: cell cycle control, differentiation, proliferation, apoptosis, autophagy, and DNA repair, among others, and thus have a role in cancer, either as oncogenes –dubbed oncomirs– or tumor suppressors (27).

Several studies show that miRNAs promote resistance to treatments in other cancer types (28); notably, they can promote radioresistance or radiosensitivity. For instance, miR-214 is upregulated in ovarian cancer, leading to PTEN mRNA degradation and PI3K/Akt activation, thus promoting radioresistance (29). miR-183-5p promotes radioresistance by decreasing ATG5 mRNA expression, interacting with downstream signaling genes from PI3K and Wnt signaling pathways, and upregulating them in colorectal cancer (30, 31). Likewise, miR-365 enhances radiosensitivity by inhibiting the CDC25A expression in non-small cell lung cancer cells, consequently improving the prognosis after IR treatment (32).

Long non-coding (lncRNAs) RNAs are 200+ nucleotide-long molecules (33), transcribed mainly by RNA pol II (34). There are recent reports of their involvement in the regulation of gene expression, metastasis, and invasion of cancer cells (33), miRNA silencing (35), apoptosis, autophagy, cell cycle regulation, and DNA repair (17, 36, 37). As the number of described lncRNAs increases (38), so does the number that regulates the biological processes mentioned above.

lncRNAs have been described in various cancer types. For example, NEAT1 is implicated in the DNA repair process by homologous recombination pathway regulating CHK1, CHK2, BRCA1, and RPA2 expression in multiple myeloma (39). FAM83H-AS1 promotes metastasis and proliferation by interacting and regulating HuR protein stability in ovarian cancer (40). ANRIL promotes proliferation, cell metastasis and inhibits apoptosis by suppressing miR-125a expression in nasopharyngeal carcinoma cells (41). POU3F3 inhibits autophagy signaling by decreasing SMAD4 in colorectal cancer and is involved in cell proliferation and migration (42). Finally, upregulated WTAPP1 promotes invasion and migration in non-small cell lung cancer by interacting with HAND2-S1 and decreasing its expression (43).

Most interestingly, these two classes of ncRNAs can interact with each other, adding to the complexity and importance of their regulation on mRNAs. Several lncRNAs have regions complementary to miRNA sequences that compete for their binding with the target mRNA. This binding sequesters miRNAs to complementary lncRNAs and prevents them from binding to their mRNA targets, turning lncRNAs into miRNA sponges, effective positive mRNA regulators (44, 45).

Interactions of this kind have been reported in diverse biological processes. lncRNA PCAT1 downregulates miR-128 in cervical cancer, promoting proliferation, migration, invasion and thus decreasing radiosensitivity (46). LncRNA *lnc-RI* competitively binds with miR-4727 regulating Non-Homologous End Joining (NHEJ) through LIG4 mRNA stabilization, affecting cell cycle and radiosensitivity in colorectal cancer (47). LncRNA TRPM2-AS in gastric cancer serves as a sponge for miR-612, promoting radioresistance by upregulation of the DNA double-strand break repair protein FOXM1 (48). Several interactions like these have been reported in TNBC. For instance, lncRNA WEE-AS1promotes proliferation by downregulating miR-32-5p (49), while LINC00173 downregulates miR-490-3p and promotes a more aggressive phenotype (50). Recently, Yuan and colleagues identified MAL2 and NEAT1 as key miRNA regulators in TNBC through an in silico approach (51).

Both miRNAs (52) and lncRNAs (53) have been employed as radiotherapy response biomarkers; however, more research is needed to understand their role in radioresistance fully. A complete grasp of this process and its elements will provide a knowledge base for increasing radiotherapy’s effectiveness in breast and other cancer types. This review describes the different miRNAs, lncRNAs, and their associations that regulate resistance against ionizing radiation-based therapies in breast cancer. We found that these ncRNAs are mainly involved in DNA damage response, but they are also involved in cell death, cell cycle regulation, and other functional aspects. In the following sections, we summarize the currently described ncRNAs involved in the alteration of these processes.

## Methods

We searched the Medline database for journal articles in English, published from 2001 to 2021, using combinations of the following keywords: lncRNA, miRNA, breast cancer, radiotherapy, radioresistance, and radiosensitivity.

We obtained 45 articles reporting the diverse roles of ncRNAs in radioresistance. We thoroughly read each paper and extracted data about the type of ncRNAs, targets, and pathways involved in cell radiosensitivity or radioresistance mechanisms, the type of cell line used in both *in vivo* or *in vitro* assays; subsequently, we constructed three ncRNA interaction networks using Cytoscape, available at NDEx. (https://www.ndexbio.org/#/). These networks correspond to those processes most regulated by ncRNAs: DNA damage, apoptosis and autophagy, and cell cycle.

## ncRNAs Involved in DNA Damage

DNA damage response system (DDR) is a complex network comprising several processes to locate and correct DNA damage to maintain genomic integrity. This extensive network includes mechanisms for damage detection, signal transduction, DNA repair tolerance processes, and cell cycle control. For detailed descriptions of the proteins that participate in these processes, please refer to Giglia-Mari et al. (54).

DNA is an intrinsically reactive molecule and is highly susceptible to damage or chemical alterations due to endogenous processes and factors, such as replication errors, spontaneous deamination of bases, oxidative damage by ROS and formation of abasic sites; or by exogenous agents, for example, DNA breaks by IR, alkylation of bases by chemical agents, modification of bases by ultraviolet (UV) radiation, among others (55, 56). The main repair mechanisms for these damages are nucleotide excision repair (NER), base excision repair (BER), homologous recombination (HR), non-homologous end junction (NHEJ), and mismatch repair (MMR). These processes are extensively explained by Christmann et al. (57).

DNA double-strand breaks (DSB) are the most predominant and damaging lesions caused by IR (58). The most common DSB repair mechanisms are the Homologous Recombination (HR) and the Non-Homologous End Joining (NHEJ) pathways (59). The cell cycle phase determines the triggering of one or the other, but in both cases, they require the intervention of other DDR proteins (54). In addition to the proteins involved in DDR, many ncRNAs are essential to the damage response mechanisms (60–62). Furthermore, these ncRNAs modulate the DDR elements’ activity after irradiation, promoting radioresistant or radiosensitive phenotypes (9).

### H2AX as an Indicator of Radiosensitivity

Phosphorylation of the histone variant H2AX is an early event in DDR and, thus, a reliable marker of ongoing DNA repair. However, H2AX foci decrease upon completion of the DNA repair process, so extended detection indicates radiosensitivity (63). The effect of multiple ncRNAs that target DDR proteins can be assessed through H2AX detection.

P. Zhang and collaborators (64) found BC cells that overexpress miR-205 exhibit persistent H2AX foci, indicating their low capacity to repair damage after IR. The authors suggest that ZEB1 mediates the effect of miR-205 by partially restoring repair. They demonstrated that miR-205 inhibition increases the expression levels of ZEB1 and Ubc13[u1] [u2]. Therefore, miR-205 radiosensitizes BC cells by inhibiting HR by targeting ZEB1 and Ubc13.

Similarly, Mei and colleagues (65) reported that BC cells transfected with the miR-15 family of mimics showed persistent higher levels of gamma-H2AX after irradiation, indicating unrepaired DNA damage. It is well-known that gamma-H2AX foci decrease shortly after radiation; these authors suggest that the miR-15 family be involved in inhibiting DNA repair, thus acting as radiosensitizers.

Masoudi-Khoram et al. (66) used gamma-H2AX and RAD51 as markers to evaluate DNA damage by IR in two BC-derived cell lines. They found that RAD51 expression increased post-radiation while gamma-H2AX expression reached a peak 4 hours after irradiation and then rapidly decreased. They identified miR-16-5p as a possible important mediator of radiation response and suggested that miR-16-5p could promote radiosensitive breast cancer cells to IR.

In a study with diverse cancer-derived cell lines, Koo and colleagues demonstrated that miR-200c overexpression in the breast cancer cell line, MDA-MB-468 provoked an increase of gamma-H2AX foci and prolonged focus formations after irradiation. This effect was associated with a discernible downregulation of p-DNA-PKcs involved in NHEJ repair (67).

Lin et al. (68) found that overexpression of miR-200c enhanced IR-induced DNA strand breaks in BC cell culture. They found a correlation between increased miR-200c expression and the presence of H2AX foci. Years later, Wang et al. (69) discovered that lncRNA LINC02582 is a downstream target of miR-200c. LINC02582 interacts with USP7 to deubiquitinate and stabilize CHK1, a critical effector in response to DNA damage that facilitates DNA repair, promoting radioresistance (70). However, their results demonstrated that miR-200c expression reduced the CHK1 protein level since it targets LINC02582. They suggest the miR-200c/LINC02582/USP7/CHK1 signaling axis as a potential target to improve breast cancer response to radiation therapy.

In another study with diverse cancer cell types, including BC cells, Lee et al. (71) described miR-7 as a radiosensitizer. Its overexpression causes downregulation of EGFR, AKT, ERK, and STAT3. They inhibited miR-7, which led to positive regulation of EGFR and its downstream effectors to validate these results. Besides, they reported that ectopic overexpression of the miR-7 caused marked prolongation of radiation-induced gamma-H2AX foci formation. The authors associated this phenomenon with a decrease in DNA-PKcs phosphorylation with an activated EGFR-associated signaling pathway.

Zhang et al. (72) found a positive correlation between the expression of LINP1, Ku80, and DNA-PKcs after IR and identified that the lncRNA LINP1 binds Ku80 and DNA-PKcs, promoting radioresistance. They hinted that DSB repair is enhanced by LINP1 across the NHEJ pathway due LINP1 to providing a scaffold for Ku80 and DNA-Pkcs. The authors confirmed this by measuring DNA damage through gamma-H2AX. When LINP1 was removed, gamma-H2AX foci were more persistent. Besides, they discovered that activation of EGFR upregulates LINP1 transcription through activation of the RAS-MEK-ERK pathway; in this manner, cells with EGFR activation improve DNA repair through the LINP1/Ku80/DNA PKcs axis. Also, they identified a negative feedback mechanism where p53 and miR-29 are involved. P53 regulates the expression of miR-29 directly, and, in turn, this negatively regulates LIPN1; this is an uncommon miRNA-lncRNA interaction since lncRNAs sponge miRNAs in most of the currently described instances.

### ncRNAs That Target HR Proteins

RAD51, catalyzes the strand transfer between a broken sequence and its homolog to re-synthesize the damaged region (73). Gasparini et al. (74), demonstrated that miR-155 effectively reduces HR repair by targeting RAD51 directly; thus, miR-155 contributes to increased sensitivity to IR. These findings were established both *in vivo* and *in vitro*. They found that miR-155 overexpression is associated with lower RAD51 expression; besides, they found a higher survival rate in a TNBC patient cohort due to the anti-correlation between miR-155 overexpression and its target RAD51.

Another study (75) demonstrated that miR-302a downregulation confers radioresistance and that restoration of its expression sensitizes breast cancer cells to radiotherapy since miR-302a targets RAD52, an essential participant in HR repair, and AKT1 (76). Chai et al. (77) show that miR-185 was downregulated in radioresistant BC cells and that there is an inverse correlation with the expression of AKT1 and RAD52. Besides, induced overexpression of miR-185 decreases the expression of AKT1, RAD52, and Bcl-2.

In another work that involved alteration of HR participants, Troschel and collaborators (78) reported that miR-142-3p can sensitize breast cancer cells to radiotherapy by downregulating BRCA1 and BRCA2, two proteins that mediate DSB repair by HR. BRCA1 and BRCA2 play a role as mediators of recombination, promote ssDNA resection, and are believed to be required for subnuclear assembly of RAD51 (79).

Another workgroup found that miR-671-5p was inversely correlated with FOXM1. Through HCR assay, the authors measured the DNA repair capability in breast cancer cell lines. In cells with miR-671-5p inhibited after IR, the HCR activity was significant compared to the control, and FOXM1 expression also increased. Their western blot results showed that miR-671-5p suppressed the expression of genes downstream from FOXM1 involved in the DNA repair pathway; these are RAD51 and BRIP1, the latter contributes to the DNA repair function of BRCA1 (80). Thus, their results hint that miR-671-5p radiosensitizes breast cancer cells by targeting the FOXM1 target, affecting downstream genes involved in DNA repair (81).

### ncRNAs That Target Other DDR Proteins

The lncRNA HOTAIR has recently emerged as a multifunctional regulator. Quian et al. (82) demonstrated that HOTAIR could induce resistance to radiotherapy in breast cancer cells. They found that the Ku70 and Ku80 proteins, DNA-PKs, and ATM were upregulated due to HOTAIR overexpression, thus promoting repair and reducing IR sensitivity. In response to DSB, Ku70 and Ku80 associate with broken end chains and then recruit DNA-PKcs to the damage sites, i.e., Ku proteins act as a scaffold for other proteíns that participate in the NHEJ pathway (83, 84).

Surprisingly, it was reported that miR-139-5p modulated resistance to radiation in breast cancer by affecting multiple genes involved in DDR. Five of its six confirmatory targets have roles in diverse DDR pathways essential for post-radiation damage repair. These pathways include microhomology-mediated end-junction (MMJE) with POLQ and XRCC5, BER in which miR-139-5 targets POLQ, NHEJ with XRCC5, HR for RAD54L. Additionally, it regulates DNA topology during repair targeting TOP2A and TOP1 and seems to have a ROS defense role by targeting MAT2AT (85).

**Figure 1:**
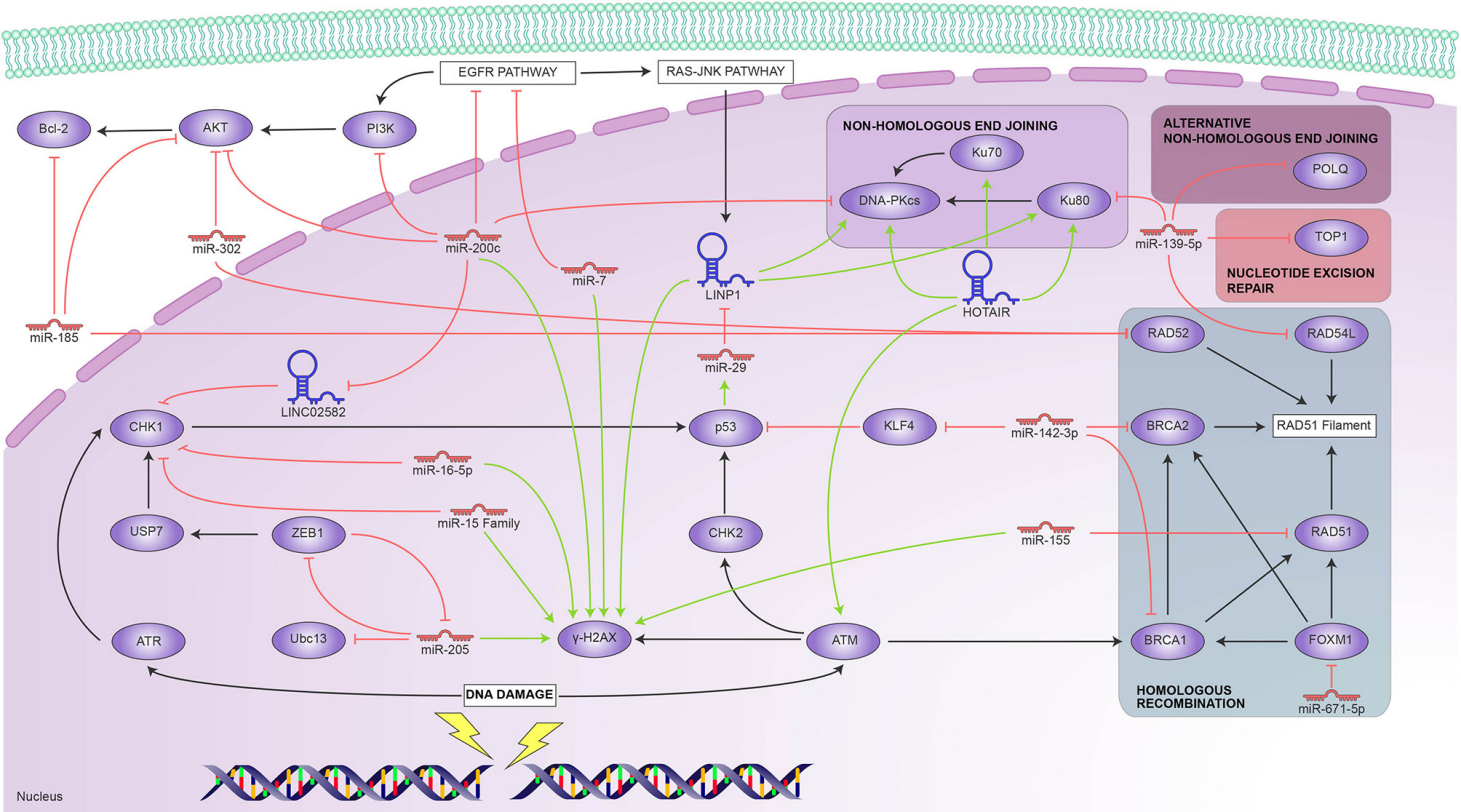
Reported ncRNAs that regulate DNA damage response and their targets. Green arrowheads represent positive regulation and red bars, negative regulation.

The above findings are summarized in **Figure 1**, which shows the main elements involved in DDR and the ncRNAs that reportedly regulate them. We found it interesting that only one report involved ATM, one of the primary DNA-damage sensors; ATM responded to HOTAIR overexpression, as did the Ku proteins. There are, however, reports of several ncRNAs that target virtually every downstream pathway—notably, miR139-5p targets proteins that participate in HR, NER, and NHEJ. miR-200c is also multifunctional in DDR; it targets CHK1 through LINC02582 and the DNA PKcs involved in NHEJ. This repair pathway is also targeted by the LINP1 lncRNA, itself regulated by P53 through miR-29; conversely, our search only yielded reports of HR being regulated by miRNAs, such as miR-155 and miR-142. All these ncRNAs are potential radioresistance markers and attractive targets towards induced radiosensitivity.

## ncRNAs Involved in Apoptosis and Autophagy

Dysregulation of cell death plays a key role during carcinogenesis. Multiple alterations occur within apoptotic pathways leading to an overall reduction of apoptosis in tumor cells and the rise of apoptosis-resistant phenotypes (86, 87).

### Apoptosis

Apoptosis is the most common form of controlled cell death, in which the cell gradually collapses and ultimately dies. It can be triggered by the intrinsic pathway, initiated by either the absence or excess of growth factors, hormones, and cytokines, or by the extrinsic pathway, set off by interaction between death ligands and death receptors such as those from the Tumour Necrosis Factor (TNF) family. For further detail on apoptosis and its effectors, please refer to Cao & Tait (88).

Several ncRNAs regulate the apoptotic response to IR in BC. Yu and colleagues (89) observed an association between miR-144 overexpression and cell survival after irradiation. Subsequent experiments revealed that miR-144 overexpression increased Bcl2 levels and inhibited the pro-apoptotic protein Bax and caspase activity; meanwhile, PTEN and pAkt showed aberrant expression levels, suggesting that miR-144 regulate the radiation-induced apoptotic response by targeting the PTEN/Akt signaling pathway.

Overexpression of the multifunctional lncRNA HOTAIR was also observed in BC cells following irradiation; high expression of this lncRNA has been associated with radioresistance acquisition, even though the exact role of HOTAIR in this process remains unclear. In-vitro experiments showed alterations in the proliferative and apoptotic cells ratio, altered Akt expression, and downregulation of the pro-apoptotic Bad protein. These findings suggest that HOTAIR induces radioresistance by inhibiting apoptosis *via* the PI3K/Akt-Bad signaling pathway (90). A more recent study suggests another possible mechanism for HOTAIR-induced radioresistance. Knockdown of HOTAIR resulted in an increase of radiation-induced apoptosis, DNA damage, cell cycle arrest, and an upregulation of miR-218. Since miR-218 upregulation promoted cell apoptosis, this data suggests that the HOTAIR-miR-218 axis plays a critical role in radiation-induced apoptosis (36).

Other authors found an upregulation of the lncRNA CCAT1 in radioresistant BC tissues where miR-148b was found to be downregulated. The interaction between CCAT1 and miR-148b was confirmed through luciferase reporter assay. Downregulation of CCAT1 increased radiosensitivity through inhibiting proliferation and promoting apoptosis, implying that the CCAT1-miR148b interaction regulates the acquisition of radioresistance in BC cells (91).

On the other hand, ncRNAs have also been found to sensitize BC cells to radiotherapy by inducing apoptosis. Zhu and colleagues (92) observed that the upregulation of miR-195 enhanced radiosensitivity in BC cells *via* increasing radiation-induced apoptosis by downregulation of Bcl2. More recently, Chai and colleagues (77) reported downregulation of miR-185 in radioresistant BC cells; conversely, overexpressed miR-185 radiosensitized BC cells. miR-185 overexpression led to Bcl2 downregulation, thus identifying Bcl2 as a downstream target of miR-185. Further experiments showed that Bcl2 silencing radiosensitized BC cells, confirming the role of the miR-185-Bcl2 axis in radioresistance.

In another study, miR-122-3p overexpression was found to sensitize BC cells to ionizing radiation. It was also found that miR-122-3p overexpression induced apoptosis after irradiation while suppressing migration and invasion. Additionally, the aberrant expression levels of PTEN/PI3K/AKT and EMT pathways proteins suggest that miR-122-3p might control radiation-induced apoptosis by regulating the PTEN/PI3K/AKT pathway (93).

### Autophagy

Autophagy is a set of adaptations usually aimed at avoiding cell death by sequestering and recycling a portion of the cytoplasm and organelles. Still, it can be triggered to remove damaged or senescent organelles to maintain energy balance or as a result of nutrient deprivation, ultimately leading to cell death. Descriptions of the involved proteins and their functions can be found in reviews such as those by Doherty & Baehrecke (94), Kim & Lee (95), and Maiuri et al. (96). Autophagy plays a dual role during carcinogenesis, leading to cell death or promoting cell survival *via* inhibiting apoptosis (97).

Several workgroups have demonstrated that ncRNAs play a role in the regulation of autophagy in BC after irradiation. Yi and colleagues (98) observed that the overexpression of miR-199a-5p in MCF7 cells inhibited radiation-induced autophagy. Inhibition of Beclin1 and DRAM1 due to miR-199a-5p was also observed, identifying them as downstream targets and suggesting a potential mechanism for radiation-induced autophagy. However, experiments in the MDA-MB-231 cell line showed that miR-199a-5p overexpression upregulated Beclin1 and DRAM1, promoting radiation-induced autophagy. Further experiments showed that miR-199a-5p regulates cell cycle arrest after IR; additionally, it altered the radiation response of BC after IR. This evidence confirms a role for miR-199a-5p in radiation-induced autophagy through a still undetermined underlying molecular mechanism.

In the same way, miR-200c sensitized BC cells to IR. miR-200c overexpression inhibited radiation-induced autophagy in BC cells; moreover, UBQLN1, a protein associated with promoting autophagosome formation, was identified as a downstream target of miR-200c. This finding suggested that miR-200c enhances radiosensitivity in BC cells by suppressing radiation-induced autophagy through the regulation of UBQLN1 (99).

Luo and colleagues (100) found that the overexpression of miR-129-5p sensitized BC cells to IR, while autophagy acted as a protective response. Subsequently, miR-129-5p was found to inhibit autophagy during the early stages of autophagosome formation, promoting apoptosis. HMGB1 was identified as a potential downstream target for miR-129-5p using online databases. HMGB1 knockdown reduced cell survival and radiation-induced autophagy, suggesting that miR-129-5p may radiosensitize BC cells by inhibiting radiation-induced autophagy *via* directly targeting HMGB1.

**Figure 2:**
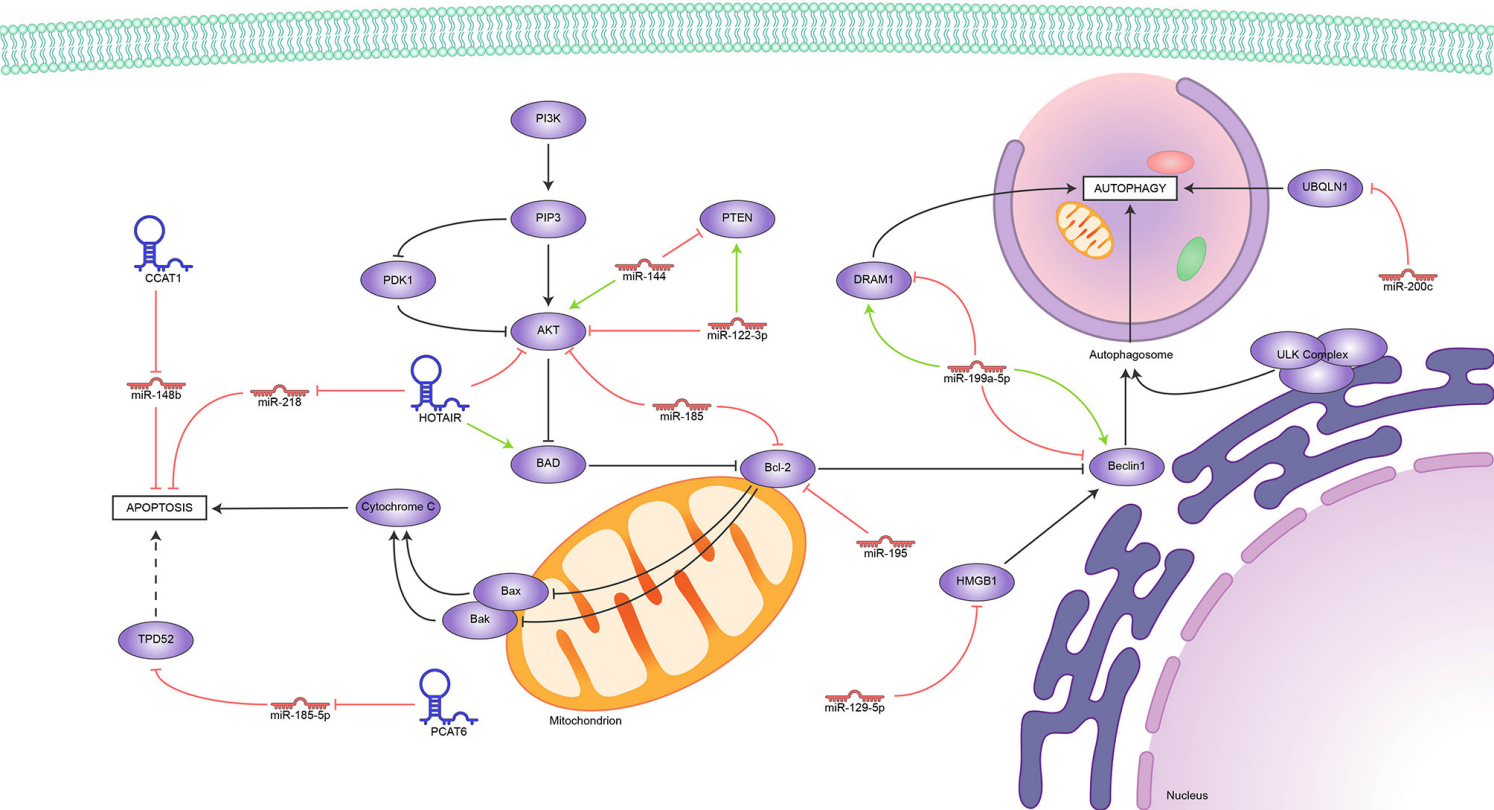
Reported ncRNAs that regulate apoptosis, autophagy, and their targets. Green arrowheads represent positive regulation and red bars, negative regulation.

Unsurprisingly, we found reports of several lncRNAs that induce radioresistance by blocking apoptosis and others that perform the opposite function, all of them represented in **Figure 2**. So far, the evidence appoints Bcl2 as the hub of this regulation; it is upregulated indirectly by miR144 and downregulated by miR-185 and miR-195. Meanwhile, the HOTAIR-miR-218, CCAT1-miR148b, and PCAT6-miR-185-5p axes block apoptosis through mechanisms still under study. Conflicting reports on the role of miR-199-5p show how much more there is to know about the role of ncRNAs in the delicate balance between apoptosis and autophagy in tumor development.

## ncRNAs Involved in Cell Cycle

The equilibrium between cell proliferation and death is tightly controlled by the cell cycle, a complex regulatory network that progresses through alternating cell growth, subcellular component synthesis, and cell division phases. Cell cycle progression is regulated primarily by a family of proteins called cyclins that bind and activate their effector counterparts, cyclin-dependent kinases, or CDKs. In an undisturbed cell, timely cyclin expression activates the necessary CDKs, which, in turn, phosphorylate multiple targets that control phase-specific processes. Cyclin D is expressed from early G1 and activates CDK6, both cyclin E in late G1-S and cyclin A in early S-G2 phase activate CDK2, and cyclin B activates CDK1 in G2-M. For additional details on these and other cell cycle regulators and their alterations in cancer, please refer to Foster (101).

Cell cycle checkpoints are, essentially, fail-safe mechanisms that prevent cell cycle progression in response to stimuli such as cell overgrowth, suboptimal chromosome segregation during mitosis, and, notably, DNA damage (102). The ATM/ATR–p53 signaling pathway, part of the DDR, induces G1 or G2 arrest, allowing for DNA repair prior to replication or preventing the cell from undergoing mitosis with a set of altered chromosomes, respectively. However, these mechanisms are dysregulated in cancer cells and let cells with accumulating mutations proliferate (103). In this way, several ncRNAs are upregulated in BC and BC-derived cell lines, associated with a radioresistant phenotype both in patients and cell cultures, suggesting active participation of ncRNAs in the modulation of the response to radiotherapy.

### G1/S Checkpoint

Zhang and collaborators (104) found that LINC00963 expression led to the upregulation of the cell cycle regulatory proteins cyclin D1 and CDK6, leading to higher p27 levels and cell cycle progression. Furthermore, elevated LINC00963 expression was significantly associated with tumor size and metastasis. These authors searched for potential miRNA targets and found that LINC00963 sponged miR-324-3p and upregulated ACK1, which belongs to a family of non-receptor-tyrosine-kinases and functions as a driver of tumor progression.

Liu and colleagues (17) found a strong association between cell survival *in vitro* and increased LINC00511 expression, besides its significant over-expression in BC patients. Subsequent in-vitro experiments correlated its expression with radioresistance and a higher cell proliferation rate. These authors performed a bioinformatic search for miRNA targets and found that LINC00511 sponges miR-185 upregulating STXBP4. This protein has been proven to promote cell cycle progression through TP63 activation (105).

On the other hand, some ncRNAs were recently shown to increase radiosensitivity. For instance, the multifunctional lncRNA HOTAIR increased its expression in BC cells upon radiation exposure. Experimental HOTAIR knockdown increased DNA damage and led to cell cycle arrest. It was also observed that HOTAIR exerted its radiosensitizing effect through the downregulation of miR-218, although the corresponding upregulated target is still to be elucidated (36).

### G2/M and Spindle Checkpoints

Mei and colleagues found that miR-15a, 15b, and 16 influence radiosensitivity of MCF7 and MDA-MB-231 breast cancer cells, observable through the enhanced duration of H2AX foci and release of the G2 arrest induced by radiation. They demonstrated the interaction between these miRNAs and the cell cycle regulator WEE1 and CHK1 mRNAs through luciferase assays, but they did not find the dramatic reduction they expected at the protein level, hinting at a more complex mechanism (65). In a differential miRNA expression study, miR-16-5p was upregulated in correlation with radiosensitivity in the radiosensitive T47D and the radioresistant MDA-MB-231cell lines. Through bioinformatic analyses, these authors predicted its interaction with targets such as WEE1, Chk1, and CDC27 (66). miR-16-5p had been previously observed to inhibit proliferation in prostate (106) and breast (107) cancers by targeting AKT.

Low CDC27, a component of the anaphase-promoting complex, is a radioresistance marker in TBNC (108). According to a study in MDA-MB-231 cells, this affects the corresponding miR-27a overexpression, which targets CDC27 and increases cell proliferation even under ionizing radiation (109).

**Figure 3:**
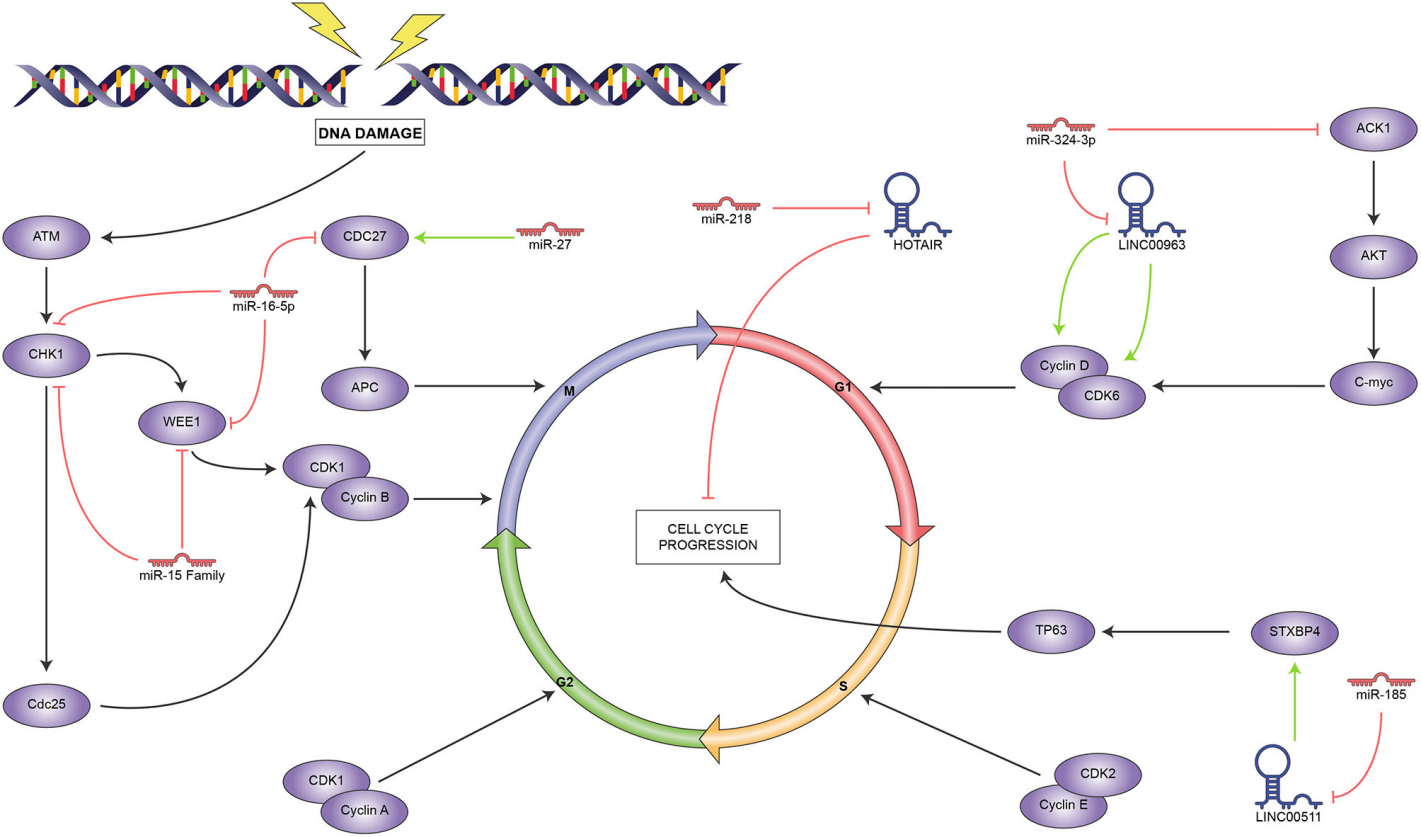
Reported ncRNAs that regulate cell cycle and their targets. Green arrowheads represent positive regulation and red bars, negative regulation.

According to these reports, ncRNAs mainly regulate the spindle checkpoint, as it is strongly controlled by the miR-15 family and the closely related miR-16; meanwhile, LINC00963 regulates the G1/S checkpoint, as seen in **Figure 3**. Interestingly, we found no reports of ncRNAs that influence the S or G2 phases, which leaves ample room for research in this area, given the importance of cell cycle control in cancer. In this regard, the mechanisms employed by HOTAIR and LINC5011 to control cell proliferation are still to be determined.

## Other ncRNA Targets

Besides those reviewed in the preceding sections, our search yielded reports on ncRNAs that induce either radioresistance or radiosensitivity by regulating processes such as cell signaling, metabolism, and inflammation, although these were not as abundant. We briefly summarize them in this section and **Table 1**, hoping to encourage further research in these aspects.

**Table 1:** Reported ncRNAs that regulate cell signaling, cell metabolism, and inflammation.

ncRNA	Target	Pathway	Reference
**ncRNA promoting radiosensitivity**			
**miR-634**	STAT3	JAK-STAT signaling pathway	Yang et al. (110)
**miR-223**	EGFR	EGFR signaling pathway	Fabris et al. (111)
**miR-7**	EGFR, Akt	EGFR signaling pathway, PI3K-AKT signaling pathway	Lee et al. (71)
**miR-125b**	c-JUN	JNK signaling pathway	Metheerairut et al. (112)
**miR-671-5p**	FOXM1	Cellular senescence	Tan et al. (81)
**Let-7**	–	Embryonic development	Wang et al. (113)
**miR-22**	Sirt1	AMPK signaling pathway	Zhang et al. (114)
**miR-770-5p**	PBK (PDZ-binding kinase)	–	Lee et al. (115)
**ncRNA promoting radiorensistance**			
**miR-124**	STAT3	JAK-STAT signaling pathway	Fu et al. (116)
**miR-122**	ZNF611, ZNF304, RIPK1, HRAS, Dusp8, TNFRSF21	TNF signaling pathway, RAS-MAPK signaling pathway	Pérez-Añorve et al. (117)
**miR-34**	p53	p53 signaling pathway	Kato et al. (118)
**miR-33a**	ABCA1/ABCG1	Lipid metabolism	Wolfe et al. (119)
**HOTAIR**	miR-449b-5p, HSPA1A	JNK signaling pathway	Zhang et al. (120)
**miR-210, miR-10b, miR-182, miR-142, miR-221, miR-21, miR-93, miR-15b**	–	–	Grinan-Lison et al. (52)
**miR-620**	HPGD/PGE2	Metabolism of prostaglandins	Huang et al. (121)
**PCAT6**	miR-185-5p, TPD52	Membrane traffic	Shi et al. (122)
**NEAT1**	NOQ1	Oxidative stress	Lin et al. (123)
**miR-668**	IkB-alfa	NF-Kappa B signaling pathway	Luo et al. (124)

### Cell Signaling

STAT3 is a transcription factor that regulates gene expression in response to several stimuli such as growth factors and interleukins. In breast cancer, it regulates several target oncogenes and participates in cancer progression, metastasis, apoptosis, and resistance to therapies (125), and it is targeted by ncRNAs modifying the response to radiotherapy in breast cancer.

miR-124 was negatively regulated in HER2-positive breast cancer cells; this miRNA directly targets STAT3, which regulates HER2 expression. So, miR-124 overexpression caused STAT3 downregulation and enhanced radiotherapy response by increasing cell death. The weak miR-124 expression could enhance STAT3 expression and promote radioresistance in HER2-positive breast cancer (116). Similarly, Yang and coworkers (110) observed that miR-634 was significantly decreased in breast cancer cell lines upon radiation. A miR-634 transfection assay showed an increase in apoptosis and a drastic decrease in cell survival capacity. They demonstrated that miR-634 suppresses breast cancer cells by targeting STAT3, increasing radiotherapy sensitivity.

The EGFR pathway is also associated with breast cancer progression since it regulates multiple tumorigenic processes (126). Fabris et al. (111) observed IR-induced miR-223 expression following BC mass removal. Further experiments revealed that miR-223 directly targets EGF, suggesting it may affect the activation of the EGFR pathway. Additionally, miR-223 overexpression was found to antagonize the pro-tumorigenic signals induced by wound fluids *via* negative regulation of EGF.

Overexpression of miR-122 was observed in therapy-induced radioresistant BC cells; additionally, it sensitized BC cells to IR. Contrastingly, miR-122 knockdown resulted in the acquisition of radioresistance in BC cells. Several proteins involved in diverse pathways such as the transcription factor ZNF611, the TNF pathway elements TNFRS21 and RIPK1, and the Ras-MAPK pathway mediators DUSP8 and HRAS were identified as miR-122 potential targets. These findings suggest that miR-122 may play a multifunctional role in acquiring radioresistance (117).

The JNK signaling pathway promoted cell survival in cancer by interacting with multiple pathways (127). Metheetrairut and colleagues (112) found that miR-125b sensitized BC cells to IR. miR-125b was also found to promote radiation-induced senescence in BC cells. Furthermore, c-JUN regulation by miR-125b was found to be involved in radiosensitivity in BC cells; additionally, members of the MAPK signaling pathway were targeted by miR-125b, suggesting that regulation of the MAPK-c-JUN axis by miR-125b might modulate radiosensitivity in BC cells.

Alterations in the p53 pathway play a key role during carcinogenesis (128). Kato and colleagues (118) observed radiation-induced expression of miR-34 mediated by p53 in BC cells. Furthermore, various BC cell lines showed differential miR-34 expression, and cell lines with low miR-34 levels were radiosensitive. Further experimentation revealed that miR-34 might prevent cells from radiation-induced cell death.

FOXM1 is a transcription factor necessary for many biological processes as cell proliferation, cell cycle progression, and cell differentiation. It is a master regulator of DNA damage response, and it is also associated with EMT phenotype in cancer; likewise, it promotes metastasis and tumor progression (129, 130). Tan et al. (81) demonstrated that miR-671-5p radio- and chemosensitize breast cancer cells by targeting FOXM1. They worked with 21T cells and found that miR-671-5p was decreased during breast cancer progression, contrary to FOXM1. In addition, they found that miR-671-5poverexpression reduces FOXM1 expression and affects the downstream genes involved in EMT (TGF-β and VEGF) and DNA repair during BC progression. This way, miR-671-5p inhibits cell proliferation and invasion and sensitizes breast cancer cells to IR.

### Cell Metabolism

Cholesterol regulation has proven to be involved in cancer progression (131). Wolfe and colleagues (119) found that miR-33a expression regulates HDL-induced radioresistance through targeting ABCA1. miR-33a expression was found to be lower in irradiated BC cells than in non-irradiated BC cells. Additionally, the expression of the ABCA1 protein was inversely correlated with that of miR-33a. Furthermore, knockdown of miR-33a in BC cell lines with higher miR-33a expression levels resulted in radiosensitization, whereas miR-33a mimic transfection in BC cell lines with low miR-33a expression led to the inhibition of HDL-induced radiosensitization *via* regulation of ABCA1. miR-33a was also associated with an adverse outcome in BC patients.

The Lin28/Let-7 axis, primarily active during embryonic development, regulates multiple genes involved in several tumorigenic processes (132). It may also be involved in the regulation of radioresistance in BC. Cell lines expressing higher levels of the Lin28 protein showed increased survival compared to those expressing lower levels of Lin28. Meanwhile, Lin28 knockdown showed an increase in radiosensitivity. Lin28 was also associated with the regulation of apoptosis; on the other hand, Let7 was confirmed to be directly regulated by Lin28, thus suggesting possible mechanisms for acquiring radioresistance *via* Lin28 (113).

Sirt1 is a histone deacetylase that acts as a regulator in multiple physiological processes such as cell growth, apoptosis, DNA damage and, tumor development; in addition, it promotes tumorigenesis and is upregulated in breast cancer (133–135). Zhang and collaborators (114) reported that Sirt1 is a direct target of miR-22, and their expression is antagonistic, so miR-22 improves radiosensitivity to breast cancer cells by targeting Sirt1. While miR-22 expression was downregulated in breast cancer cells after IR, Sirt1 was upregulated. However, they found that overexpression of miR-22 regulated Sirt1 expression negatively, blocking its function, such as suppressing tumorigenesis and enhancing the radiosensitivity of breast cancer.

Lee and colleagues (115) discovered that miR-770-5p radiosensitizes breast cancer cells by targeting PDZ-binding kinase (PBK). PBK is a serine-threonine kinase that has been reported to be upregulated in rapidly proliferating cells, as well as in a variety of tumors, furthermore, it was shown to promote transformation and has metastatic properties (136–138). In this study, miR-770-5p was shown to be upregulated by IR response and to be inversely correlated with PBK expression both *in vitro* and *in vivo*. Despite the oncogenic potential of PBK, the authors report that miR-770-5p can directly target PBK in radiation response, confers radiosensitivity to breast cancer.

In addition to the HOTAIR roles described in the previous sections, Zhang et al. (120) identified that it confers radioresistance to breast cancer cells through the HOTAIR/miR-449-5p/HSPA1A axis. HSPA1A is a chaperone overexpressed in a large variety of tumor lines, including breast cancer (139), and its expression exhibited a positive correlation with that of HOTAIR in irradiated breast cancer cells. Also, HOTAIR acts as a sponge for miR-449-5p, preventing it from exerting its role as a negative HSPA1 regulator, allowing the development of a radioresistant phenotype.

Cancer stem cells (CSCs) play a key role during tumor development (140). Griñán‐Lisón and colleagues (52) identified several miRNAs that may modulate some CSCs properties, such as proliferation, metastasis, and response to IR. miR-142, miR-15b, miR-210, miR-21, miR-221, miR-10b, miR-182, and miR-93, involved in multiple pathways, showed aberrant expression in various BC cell lines and patients. Their results showed that IR affected BC cell lines differentially, decreasing stemness properties in MCF7 and SKBR3 cells and increasing them in the TNBC cell line MDA-MB-231, along with miR-10b, miR-210, and miR-221 expression. Similarly, miR-10b was overexpressed in patients positive for Ki67 that received IR, while miR-210 and miR-221 were detected in the only TNBC patient with recurrence in the study.

In the same way, the lncRNA PCAT6 was found to be upregulated in TNBC tissues. Subsequent experiments showed that PCAT6 knockdown promoted radiosensitivity in BC cells by inhibiting cell survival and promoting apoptosis. miR-185-5p was later identified as a potential target for PCAT6 and shown to be negatively regulated by it. Also, miR-185-5p was found to target TPD52 directly. Knockdown of both PCAT6 and TPD52 resulted in an increased radiosensitivity in TNBC cells, indicating PCAT6 plays a role in radioresistance *via* regulating the miR-185-5p-TPD52 axis (122).

Lin and colleagues (123) found that NQO1 expression and activity were higher in radioresistant BC-derived cells, modulated by the cancer stem cell-derived NEAT1 lncRNA instead of the more traditional JNK signaling. This finding suggested that the regulation of NEAT1 in NQO1 expression was potentially mediated by suppressing NQO1-targeting miRNAs because the mRNA level was not changed in the radioresistant MDA-MB-231 cells. Still, their results suggested that NEAT might regulate the protein stability of NOQ1 in 231-RR cells through a yet undescribed mechanism. At the time of writing, this is the only report of a lncRNA associated with radioresistance exerting its function through a pathway other than gene up-regulation through miRNA sponging.

### Inflammation

Inflammation has also been related to cancer progression (141), and Huang and collaborators (121) found that ncRNAs can also regulate it. Mainly, miR-620 regulates 15-hydroxyprostaglandin dehydrogenase (15-PGDH/HPGD) negatively, which induces radioresistance driven by prostaglandin E2 (PGE2) accumulation, as 15-PGDH normally antagonizes COX-2 by degrading it.

Multiple inflammatory effects during carcinogenesis are mediated by the activation of the NF-κB pathway (142). M. Luo and colleagues (124) observed that increased expression levels of miR-668 in BC cells led to the acquisition of radioresistance while its knockdown sensitized resistant BC cells to IR. miR-668 inhibited lκBα, activating the NF-κB pathway and increasing intranuclear p65, which, in turn, enhances NF-κB binding activity. Thus, miR-668 might regulate radioresistance in BC cells by activating the NF-κB pathway.

## Concluding Remarks

Breast cancer is the most common malignancy in women and one of the leading causes of cancer death worldwide. Fortunately, radiotherapy is an effective treatment that provides local tumor control, increases survival, and reduces mortality. However, the acquisition of radioresistant phenotypes can compromise the success of therapy. In this review, we summarize the ncRNAs that participate in conferring radioresistance to breast cancer.

Recently, ncRNAs have emerged as important regulators of multiple cellular processes, and resistance to cancer treatment is no exception; we found reports of ncRNAs involved mainly in the regulation of the DDR mechanisms, followed by cell death, cell cycle, and other processes where the role of ncRNAs is studied in the same depth. A significant number of these works concerned miRNAs, although the proportion of reports on lncRNAs is likely to grow in the upcoming years since lncRNAs have a lower research age. In addition, we observed a growing trend in the number of reports of the response to IR through lncRNA-miRNA-target axes. While it is probable that most of the ncRNAs that regulate radioresistance follow this model, there are other mechanisms of action to explore.

We found of particular interest that several of the reported ncRNAs exhibit a multi-modulator capacity, targeting genes involved in various pathways. Some of them even perform dual roles inducing either radiosensitivity or radioresistance in different contexts. For example, miR-139-5p modulates five different targets involved in four different DDR pathways and, additionally, can regulate DNA topology during the repair process. miR-185 regulates AKT and BCL-2, involved in the regulation of apoptosis, and RAD52, involved in HR. The miR-15 family, comprising the closely related miR-15a, miR-15b, and miR-16, is also multifunctional; it targets CHK1, promoting the formation of the γH2AX foci, while also regulating the cell cycle by targeting WEE1. miR-200c targets multiple proteins involved in the DDR and is also involved in autophagy by regulating the UBQLN1 protein; additionally, it may participate in other major pathways such as the PI3K-AKT and the EGFR. As for lncRNAs, HOTAIR regulates proteins involved in NHEJ, and its overexpression promotes radiation-induced apoptosis, possibly by targeting the PTEN-AKT pathway and miR-218, suggesting it may be a hub where the regulation of DDR, apoptosis, and cell cycle converge.

Accumulating evidence highlights the importance of the interaction between miRNAs and lncRNA. We found reports of miRNA/lncRNA/mRNA axes with a role in BC radioresistance, such as miR-200c/LINC02582/CHK1 and HOTAIR/miR-449-5p/HSPA1A. Our findings point to an increase in this kind of report since mRNA targets are yet to be identified. For instance, miR-185 was identified as a downstream target of the lncRNA LINC00511, promoting cell cycle progression and modulating DDR by regulating unidentified mRNAs. We also found reports with solid association data between a given ncRNA and radioresistance, such as HOTAIR, NEAT1, and miR-199a-5p, whose targets and interactions are still to be determined.

On the other hand, the regulation exerted by multiple ncRNAs converges in some protein targets, evincing the importance of their roles in the acquisition of radioresistance. CHK1 was directly regulated by lncRNA LINC02582, miR-16-5p, and the miR-15 family, and WEE1 was found to be controlled by the miR-15 family and miR-16-5p. Similarly, γH2AX foci formation was induced by several ncRNAs, including miR-155, LINP1, miR-200c, miR-7, miR-16-5p, the miR-15 family, and miR-205.

Many of the ncRNAs mentioned in this review are molecular marker candidates and promising therapeutic targets. Strategies aimed at downregulating ncRNAs that confer radioresistance or re-establishing the expression of those that elicit radiosensitivity are evident possibilities for adjuvant therapies that improve the outcome of radiotherapy alone. However, to get to that point, we need to fully characterize the mechanisms these ncRNAs employ. We anticipate more profound studies on ncRNA function for the upcoming years, as more research groups aim to validate the soaring results that bioinformatic analyses yield through the use of *in vivo* and *in vitro* models. Overall, the study of ncRNAs has great potential in the development of adjuvant and targeted therapies in the quest for higher survival rates and better prognosis not only for BC patients but for all cancer patients.

## Author Contributions

EL-U conceived the review. AA-M, JS-P, CL-C, and LB-E searched and organized the information. AL-A, JS-P, LB-E, and EL-U wrote the manuscript. AL-A, JS-P, DL, and LB-E prepared the figures. CL-C contributed substantially over the discussion and revision of the manuscript. EL-U and CP-P edited the final version. All authors contributed to the article and approved the submitted version.

## Funding

This study was funded by DGAPA-PAPIIT, Universidad Nacional Autónoma de México with Grant Number IA204620, awarded to EL-U.

## Conflict of Interest

The authors declare that the research was conducted in the absence of any commercial or financial relationships that could be construed as a potential conflict of interest.

## Publisher’s Note

All claims expressed in this article are solely those of the authors and do not necessarily represent those of their affiliated organizations, or those of the publisher, the editors and the reviewers. Any product that may be evaluated in this article, or claim that may be made by its manufacturer, is not guaranteed or endorsed by the publisher.
